# Sensing Bacterial-Induced DNA Damaging Effects *via* Natural Killer Group 2 Member D Immune Receptor: From Dysbiosis to Autoimmunity and Carcinogenesis

**DOI:** 10.3389/fimmu.2018.00052

**Published:** 2018-01-25

**Authors:** J. Luis Espinoza, Mika Minami

**Affiliations:** ^1^Department of Hematology and Rheumatology, Faculty of Medicine, Kindai University, Osakasayama, Japan; ^2^Faculty of Medicine, Kindai University, Higashi-osaka, Japan

**Keywords:** natural killer group 2 member D ligands, microbiota, dysbiosis, bacterial genotoxin, immunosurveillance, inflammatory bowel disease

## Abstract

The human genome is constantly exposed to exogenous and endogenous DNA damaging factors that frequently cause DNA damages. Unless repaired, damaged DNA can result in deleterious mutations capable of causing malignant transformation. Accordingly, cells have developed an advanced and effective surveillance system, the DNA damage response (DDR) pathway, which maintains genetic integrity. In addition to well-defined outcomes, such as cell cycle arrest, apoptosis, and senescence, another consequence of DDR activation is the induction of natural killer group 2 member D ligands (NKG2D-Ls) on the surface of stressed cells. Consequently, NKG2D-Ls-expressing cells are recognized and eliminated by NKG2D receptor-expressing immune cells, including NK cells, and various subsets of T-cells. Recent pieces of evidence indicate that commensal microbial imbalance (known as dysbiosis) can trigger DDR activation in host cells, which may result in sustained inflammatory responses. Therefore, dysbiosis can be seen as an important source of DNA damage agents that may be partially responsible for the overexpression of NKG2D-Ls on intestinal epithelial cells that is frequently observed in patients with inflammatory bowel disease and other disorders associated with altered human microbiota, including the development of colorectal cancer. In this article, we discuss recent evidence that appears to link an altered human microbiota with autoimmunity and carcinogenesis *via* the activation of DDR signals and the induction of NKG2D-Ls in stressed cells.

## Introduction

The DNA damage response (DDR) is a highly efficient network of cellular pathways that play a crucial role in maintaining DNA integrity (1, 2). This surveillance system is responsible for monitoring, detecting and repairing DNA lesions, in order to prevent the generation of potentially deleterious mutations, which otherwise may result in the irreversible damage of DNA molecules, leading to cancer and other alterations in cell behavior (3, 4). The accumulation of un-repaired DNA damages in non-replicating cells, such as most of the cells in the brains or muscles of adults, is believed to contribute to the aging process in humans (5, 6). In highly replicating cells, such as hematopoietic stem cells and epithelial cells, DNA mutations that result from unrepaired DNA damages play a crucial role in malignant transformation and cancer progression (5, 7, 8). Endogenous agents capable of harming DNA, such as reactive oxygen species (ROS), lipid peroxidation products, and reactive nitrogen species (RNS) are naturally released during cell metabolic activities or hydrolytic processes (1, 9). In addition, DDR activation can be triggered by thousands of exogenous agents, including ionizing radiation, chemotherapy, virus infections, and chronic inflammation (10–13).

DNA damage response activation is controlled by three protein kinases: ataxia telangiectasia mutated (ATM), DNA-dependent protein kinase (DNA-PK), and ATM- and Rad3-related (ATR) (7, 14). Both ATM and DNA-PK are recruited by DNA double strand breaks (DSB), however, whereas DNA-PK coordinates DSB repair *via* non-homologous coupling, ATM promotes homologous recombination and cell cycle arrest at various checkpoints (14). ATR is activated in response to persistent single-stranded DNA and acts at the S-phase checkpoint (14). Upon DNA damage recognition, these kinases activate various downstream mediators including p53, CHK1, CHK2, BRCA, and H2AX, which (depending on the extent of DNA damage) may lead to cell-cycle arrest, DNA repair, senescence, or apoptosis (14–16). A key mediator of ATM signal is the checkpoint kinase Chk2, which induces G1/S checkpoint *via* Cdk2 inactivation or can block cell cycle at G2/M by preventing cyclinB1/Cdk1 complex formation (17). On the other hand, Chk1, triggered by ATR signal, activates Cdc25A phosphatase and Treslin, which induce G2 and S phase arrest (7).

Another consequence of DDR and ATM/ATR activation is the induction of cell stress molecules that are proteins expressed on the surface of damaged cells (18, 19). These stress ligands, which are usually absent in normal cells, are specifically recognized by either, the natural killer group 2 member D (NKG2D) and the DNAX accessory molecule immunoreceptors (18–20).

Natural killer group 2 member D, also known as Klrk1, is a C-type lectin-like type II transmembrane protein constitutively expressed by NK cells, activated macrophages and various T-cell subsets, such as NKT cells, CD8^+^ αβ, CD4^+αβ^, and γδ T lymphocytes (21–23). Upon engagement of specific NKG2D ligands (NKG2D-Ls), NKG2D receptor activates downstream signaling pathways resulting in effector immune responses like cytokine releases and cellular cytotoxicity (22, 24).

Recent evidence has linked various bacterial pathogens with DDR activation caused by either the direct effect of microbe produced genotoxins (25–28) or indirectly by ROS or RNS that result from the prolonged or excessive activation of host immune cells in response to certain microbes or their metabolic end-products (29, 30). This bacterial-induced DDR is not limited to highly pathogenic bacteria, since genotoxic damage induced by certain members of the commensal bacteria community (termed the “microbiota”) have been also documented (31, 32). Notably, increased expression of NKG2D-Ls on the surface of intestinal epithelial cells and its recognition by NKG2D receptor-expressing immune cells is believed to contribute to the pathogenesis of inflammatory bowel diseases (IBDs), such as ulcerative colitis and Crohn’s disease (33–35) and dysregulated gut microbiota has been etiologically linked to IBD and colorectal cancer (CRC) (27, 36, 37). In this article, we discuss recent pieces of evidence that appear to link alterations in gut microbiota with activation of the DDR. The potential effects that perturbations in this network have on the development of autoimmunity and cancer immunosurveillance are also discussed.

## NKG2D-Ls Expression

In humans, multiple families of structurally unrelated NKG2D-Ls have been identified, including the MHC class I chain-related molecules (MICA and MICB), and the UL-16 binding proteins (ULBP1, -2, -3, -4, -5, and -6) (24, 38–40). NKG2D-Ls are absent or poorly expressed on the surfaces of normal cells but they are induced under certain pathological conditions like heat shock, virus infection, oxidative stress, and malignant transformation (39, 41). The elimination of NKG2D-Ls expressing cells by NKG2D receptor-expressing immune cells is one of the underlying grounds of the concept of cancer immunosurveillance (42–44). NKG2D-Ls upregulation has been described in various human cancers, including carcinomas of the breast (45), lung, colon (46) and prostate cancer (47), as well as in melanomas (48), gliomas (49), leukemias (18), and cervix cancer (50). The expression of these molecules is tightly regulated by mechanisms that control gene transcription, mRNA stability, protein translation, and stabilization (20, 39). Intriguingly, NKG2D-Ls expression has also been documented in certain normal cells. For example, in primary bronchial epithelial cells, MICA and ULBP1-4 are detectable mainly at intracellular level, but become detectable on the cell surface when the cells are exposed to oxidative stress (51). NKG2D-Ls (mainly ULBP1) have also been detected in peripheral blood cells (52) and these proteins are particularly upregulated in activated T cells and B cells (20, 53, 54). In addition, normal gut epithelium constitutively expresses MICA, although most cells appear to express these proteins intracellularly (55). On the other hand, the aberrant expression of NKG2D-Ls has been documented in certain autoimmune diseases, especially in the damaged tissues of patients with inflammatory bowel disease (IBD) that includes Crohn’s disease (35, 56) and ulcerative colitis (33). In these disorders, NKG2D-Ls expression correlates with increased number of infiltrating NKG2D^+^ lymphocytes in the damaged tissues (33, 35). Consistent with these observations, a randomized controlled clinical trial recently showed that a single dose of an anti-NKG2D blocking monoclonal antibody, significantly reduced disease activity in patients with active Crohn’s disease (57). Despite the relatively small size of this study (78 patients) and the fact that patients with UC were not included, these encouraging data support the involvement of NKG2D/NKG2D-Ls axis in the pathogenesis or clinical course of IBD.

In patients with active Celiac disease, MICA is strongly expressed on the surface of intestinal epithelial cells and it is further upregulated by wheat gliadin, which triggers the activation of intraepithelial NKG2D^+^ lymphocytes, leading to epithelial damage and villous atrophy (55). Notably, the probiotics *Lactobacillus fermentum* and *Bifidobacterium lactis* were found to directly inhibit the toxic effects of gliadin in intestinal cells (58) and a gluten-free diet strongly downregulated NKG2D-Ls in intestinal epithelial cells and concomitantly decreased NKG2D receptor expression on infiltrating NK cells (59).

## Dysbiosis and NKG2D-Ls Expression

The community of commensal microorganisms living within the human intestines, known as gut microbiota, plays critical roles in maintaining immune tolerance and epithelial integrity (60–62).

Significant upregulation of NKG2D-Ls was observed in the intestinal mucosa of germ-free mice lacking commensal microbiota, as well as in commensal-depleted animals (ampicillin-treated mice), and low ligands expression level was restored when ampicillin treatment was stopped. Strikingly, the same study found low levels of NKG2D-Ls in animals treated with vancomycin, which was attributed to the selective propagation of the vancomycin-resistant bacterium *Akkermansia muciniphila* in mice intestines (31), indicating that NKG2D-Ls expression, at least in intestinal tissues, is largely influenced by the gut microbiota composition. Interestingly, *A. muciniphila* has been linked with anti-inflammatory protective properties against IBD (63).

The loss of microbial balance and the overgrowth of pathogenic bacteria (known as dysbiosis) is often associated with the development of autoimmune disorders and the development of CRC (62, 64, 65). Strikingly, direct microbe-induced NKG2D-Ls upregulation has been documented in human intestinal epithelial cells exposed to *Escherichia coli* strains, where the interaction between bacterial adhesin AfaE and its cellular receptor CD55 results in MICA expression (66).

Another study showed that *Pseudomonas aeruginosa* infection increased NKG2D-Ls (Rae1) in mouse airway epithelial cells *in vivo* and upregulated ULBP2 in human airway epithelial cells *in vitro*, although the mechanism of ligand induction by this pathogen is unknown (67).

*Propionibacterium acnes* was recently linked with Corpus-dominant lymphocytic gastritis (CDLG), a *Helicobacter pylori* negative entity and typically characterized by extensive infiltration of CD8^+^ T-cells in the stomach epithelium. Interestingly, *P. acnes* infection correlated with increased levels of IL-15 and the upregulation of NKG2D-Ls in the inflamed gastric epithelium. Although the mechanisms leading to NKG2D-L upregulation in this entity remains unclear, a microbe-derived stimuli, probably live *P. acnes* or microbial-derived short-chain fatty acids were proposed as triggering factors (68). Notably, CDLG frequently coexists with autoimmune disorders with altered microbiota including Celiac disease (69) and Crohn’s disease (70, 71). Moreover, propionic acid, derived from the fermentation of plant-derived dietary fiber mainly under the presence *Propionibacterium*, upregulated MICA/B in human cells including, activated T lymphocytes and different cancer lines (72). *Mycobacterium tuberculosis (M. tuberculosis)*-infected dendritic and airway epithelial cells also upregulate MICA expression *in vitro* and *in vivo*, and ligand recognition by Vγ2Vδ2 T cells expressing NKG2D receptor induces a potent inflammatory reaction (73).

Of note, albeit the above studies indicate that NKG2D-Ls upregulation is frequently observed in host cells exposed to various bacteria or their products, the molecular mechanisms of this phenomenon have not been elucidated. In addition, the exact mechanism that determines the fate of host cells exposed to dysbiosis (cell cycle arrest, apoptosis, malignant transformation or NKG2D-Ls upregulation) is currently unknown. Current data suggest that the extent of DNA damage and the resultant cellular responses determine cell fate under these stress conditions, hence in the context of dysbiosis, it is conceivable that cell fate may be dependent on the specific bacterium or group of bacteria dysregulated in the host.

As mentioned above, commensal bacteria play critical roles maintaining gut homeostasis, and this particular feature can be exploited for therapeutic purposes. The oral administration of commensal lactic acid bacteria effectively protected mice from dextran sulfate sodium-induced experimental colitis, which was attributed to the enhanced interferon-β production triggered by double-stranded RNA derived from commensal lactic acid bacteria (74). Although this study did not explore NKG2D-Ls expression on intestinal cells, it is worth mentioning that type I interferons have been shown to downregulate NKG2D-Ls expression impairing NK cells-dependent killing of target cells (38).

## Bacterial Genotoxins and DDR Activation

Various intestinal bacteria are known to release genotoxins (bacterial products capable of targeting host DNA), which together with the induction of sustained inflammation, promotes genomic instability and ultimately autoimmunity or cancer (Table 1). The first characterized bacterial genotoxin was cytolethal distending toxins (CDT), which is produced by several Gram-negative bacteria, including *E. coli, Campylobacter* sp., *helicobacter* sp., *Shigella dysenteriae, and Haemophilus ducreyi*. CDT induces DNA DSB in exposed host cells that may lead to transient cell cycle arrest or malignant transformation (32, 36, 75). Mouse liver cells exposed to CDT producing helicobacter develop dysplasia (76) and fibroblasts or intestinal epithelial cells chronically exposed to large concentrations of CDT, in the absence of immune cell clearance, show genomic instability, fail to activate DDR, and eventually become prone to malignant transformation (32).

**Table 1:** Bacterial pathogens or their products that activate DNA damage response (DDR) and may induce NKG2D ligands (NKG2D-Ls) expression in host cells.

Bacterial product	Bacterial pathogen	Target cells	Type of DNA damage	NKG2D-Ls induction	Reference
**Cytotoxin**					

AfaE-III adhesin subunit	*Escherichia coli*	Enterocyte-like Caco-2 cells	Unknown	MICA	(66)

Unknown	*Pseudomonas aeruginosa*	Airway epithelial cells	Double stranded breaks (DSBs)	MICA	(67, 77)
ExoU?		Alveolar macrophages	Caused by reactive oxygen species (ROS) released from infected host cells	ULBP2	
ExoA?					

Unknown	*Propionibacterium acnes*	Gastric epithelial cells	Unknown	MICA	(68, 72)
				MICB	
				ULBP2	
Bacterial metabolic products (propionic acid, acetate, lactate)?	*Propionibacterium* sp.	Activated T cells		MICA	
		Jurkat cells		MICB	

Unknown	*Mycobacterium tuberculosis*	Dendritic cells	DSB?	MICA	(73)
		Airway epithelial cells	Endogenous ROS	Unknown	(78)
		Macrophages	DDR/ataxia telangiectasia mutated (ATM)- and Rad3-related activation due to persistent activation of toll-like receptor (TLR) signal		

TLR ligands [LPS, Poly (IC), Zimosan]	Gram (−) bacteria	Macrophages	Endogenous ROS release	MICA	(79, 80)
	*E. coli*		Persistent activation of TLR signaling	ULBP2	
	*Listeria monocytogenes*				

CagA, VacA, γGT, urease, NapA	*Helicobacter pylori*	Gastric epithelial cells	DSB	NKG2D-Ls downregulation	(68)
			Caused by ROS released from infected host cells		

*Streptococcus pyruvate oxidase*	*Streptococcus pneumoniae*	Airway epithelial cells	DSB is caused by: 1- Endogenous ROS release 2- Bacterial-secreted hydrogen peroxide	Unknown	(81, 82)

Unknown	*Salmonella typhimurium*	Murine intestinal epithelial cells	Unknown	ULBP-like transcript-1 (MULT1)	(83)

**Genotoxin**					

Cytolethal distending toxins	*Campylobacter jejuni, Haemophilus ducreyi, Actinobacillus actinomycetemcomitans, Shigella dysenteriae, Helicobacter cinaedi, Helicobacter hepaticus, Salmonella* sp.	Intestinal epithelial cells	Single-stranded breaks	Unknown	(32, 36, 75)
			DSBs		
			DDR activation		

Colibactin	*E. coli, Klebsiella pneumoniae, Enterobacter aerogenes, Citrobacter koseri*	Intestinal epithelial cells	Interstrand crosslink, DSBs	Unknown	(84–86)

Uropathogenic-specific protein	*E. coli*	HEK293 cells	DNA fragmentation		(28, 87–89)
		HUVE cells			

Cyclo phenylalanine-proline	*Lactobacillus reuteri, Streptomyces* sp. AMLK-335, *Vibrio vulnificus, V. cholera, P. aeruginosa*, and *P. putida*	INT-407, U2OS, Huh7 cells	– ROS induction – DSB – DDR activation (ATM and downstream target CHK2)	Unknown	(90)

Pneumolysin	*S. pneumoniae*	Alveolar epithelial cells	DSB	Unknown	(91)
			DDR activation		
			ATM activation		

Another bacterial-derived genotoxin is colibactin produced by *E. coli* strains of the B2 phylogroup harboring the polyketide synthetase island (*pks*), which is also found in other *Enterobacteriaceae* members such as *Proteus mirabilis* and *Klebsiella pneumoniae* (84–86). Infection with *E. coli* harboring this genomic cluster generates DSB leading to DDR activation, cell cycle arrest and genomic instability (36, 85). Notably, *E. coli* harboring *pks* are frequently detectable in patients with IBD, as well as in patients with CRC, suggesting that *pks* is directly related to disease pathogenesis (26, 92).

*Escherichia coli* uropathogenic-specific protein (Usp) is another bacterial toxin that induces genotoxic stress and activates DDR in exposed cells. This genotoxin is produced by *E. coli* strains associated with pyelonephritis, prostatitis, and bacteremia (87, 88). Purified Usp cleaves linearized naked DNA *in vitro* and causes DNA fragmentation in mammalian cells (28).

Interestingly, compared with normal intestinal samples where toxin-producing bacteria constitute a minority of the commensal microbiota, human CRC tissues contain a high expression of these microorganisms (93).

Cyclo phenylalanine-proline (cFP) is other genotoxin produced by various bacteria such as *Lactobacillus reuteri, Streptomyces* sp. AMLK-335, *Vibrio vulnificus, V. cholera, P. aeruginosa*, and *P. putida*. Mammalian cells like INT-407, U2OS, and Huh7 cells exposed to cFP develop DSB and eventually activate ATM-CHK2 (90).

Pneumolysin, a toxin produced by *Streptococcus pneumoniae* and a key virulence factor against host cells, induces DSBs and ATM-mediated H2AX phosphorylation in epithelial alveolar cells. Consequently pneumolysin-exposed cells undergo cell cycle arrest and apoptosis, although the induction of NKG2D-Ls was not investigated in this study (91). Interestingly, pyruvate oxidase, another cytotoxin released by *S. pneumoniae*, induces DSBs and contributes to pneumolysin release (81).

Bacteria may also trigger DDR activation by inducing the expression of enzymes that enhance ROS in host cells, which lead to DNA damage or the induction of chronic inflammation (36, 94, 95). Bacterial-induced DNA damage can be further amplified by ROS released from immune cells at sites of chronic inflammation since inflammatory especially macrophages and neutrophils constitute a constant source of ROS, RNS, and cytokines that can develop in response to dysbiosis (94). Granuloma formation associated with *M. tuberculosis* infection was recently linked to the persistent inflammatory signals mediated by toll-like receptor (TLR) signals, which promotes macrophage polyploidy by regulating DDR signals *via* ATR activation (78). Although this study did not assess the expression of NKG2D-Ls in polyploidy macrophages, previous studies have shown NKG2D-Ls upregulation in macrophages exposed to bacterial-derived products *via* TLR signal activation (79, 80).

**Figure 1:**
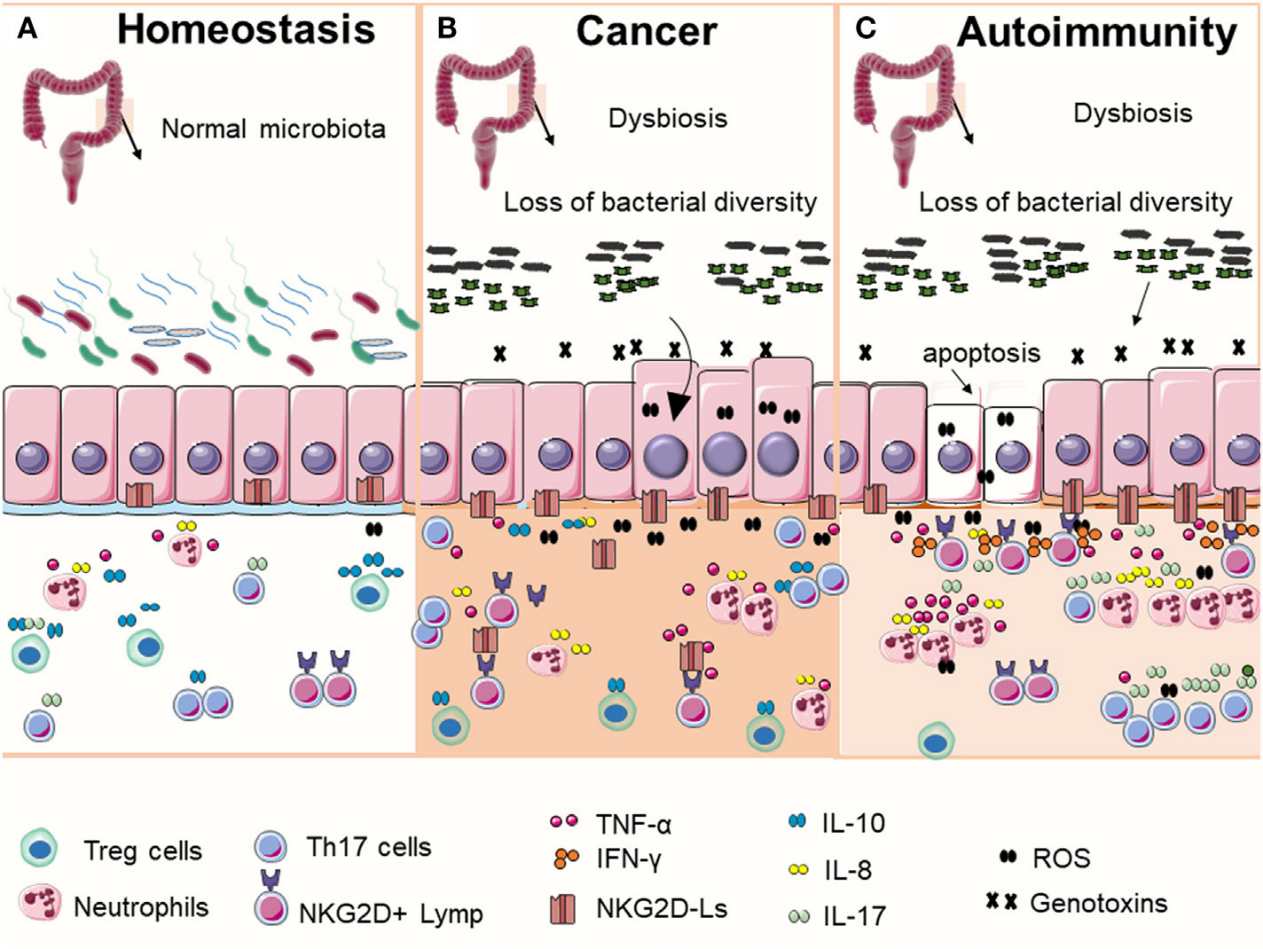
Commensal bacteria play an important role in maintaining gut homeostasis **(A)**. In normal condition, intestinal epithelial cells express low levels of NKG2D ligands (NKG2D-Ls) (mostly intracellular) and beneficial bacterial contribute to immune education and help to maintain immune tolerance by promoting the induction and accumulation of regulatory T cells (Treg cells). In the context of microbial imbalance (dysbiosis), pathogenic bacteria may release genotoxins that generate DNA damage in host cells. DNA damage response (DDR) is then activated, which may lead to cell cycle arrest, apoptosis, or NKG2D-Ls induction in exposed cells. These events, together with the sustained immune activation in response to dysbiosis may eventually contribute to the development of autoimmune disorders or malignant transformation. Although the precise mechanisms that determine why cells take one of these two contrasting cell fates are unclear, current data appear to suggest that **(B)** bacteria-induced DNA damage in cells with failed DDR (caused by mutations or by inhibitory factors secreted by bacteria) may result in the survival and proliferation of cells with unrepaired DNA, increasing the risk of malignant transformation. Transformed cells may release or shade NKG2D-Ls that impairs NKG2D receptor-mediated functions leading to failed immune surveillance and tumor growth. **(C)** Alternatively, the chronic exposure to genotoxin-secreting bacterial in host cells with fully functional DDR may result in NKG2D-Ls overexpression *via* ataxia telangiectasia mutated activation leading to increased risk of autoimmunity.

In addition, convincing evidence have established a link between infections with certain bacteria such as *E. coli, Bacteroides fragilis*, and *Fusobacterium nucleatum* with the development of CRC (26), indicating that dysbiosis may either cause carcinogenesis or autoimmunity. In some circumstances, bacterial can interrupt the DDR activation in host cells, thus allowing the cell cycle to progress in cells with unrepaired or in repaired with errors DNA resulting in mutations of critical genes associated with malignant transformation (26, 32). It is conceivable that the chronic exposure to genotoxin-secreting bacteria in host cells with fully functional DDR may result in NKG2D-Ls overexpression, *via* ATM activation, which ultimately increases the risk of autoimmunity. Conversely, in host cells with failed DDR, DSB may result in the survival of cells with unrepaired DNA and elevated risk of malignant transformation (Figure 1). This assumption is supported by the observations that epithelial cells exposed to *Chlamydia trachomatis*, a bacteria associated with cervical and ovarian cancer development, undergo DNA damaged but fail to activate DDR, due to bacterial-induced impaired DNA repair. Consequently, infected cells continue to proliferate in an environment favorable for malignant transformation (96).Thus, the NKG2D/NKG2D-Ls axis maintains a delicate immune equilibrium, which is crucial for cancer immunosurveillance but that, under certain conditions, it can eventually promote autoimmunity (97).

Despite the above studies have consistently shown that bacterial genotoxin can activate DDR, unfortunately, none of them explored the potential NKG2D-Ls upregulation in host cells exposed to those bacterial products; therefore, further studied are needed to elucidate the immunological impact of genotoxins.

## Concluding Remarks

Cells are continuously exposed to hostile environmental stressors, including extremes of temperature, toxins, and oxygen or nutrient deprivation. As a result, cells have evolved a wide range of molecular changes and stress responses to minimize damage which, depending on the severity and duration of stress confronted, can range from the activation of survival-promoting pathways to eliciting cell senescence or programmed cell death (98). During the last few years, remarkable progress has been made in our understanding of the molecular mechanisms of DDR activation and its role in various cellular processes like aging and cancer development, and it has become apparent that the immune system constitutes an important component of the cellular response to DNA damage stressors (99). In this regard, the recognition of NKG2D-Ls overexpressed on the surface of host cells as a consequence of DDR activation constitutes one of the mechanisms by which the immune cells expressing the NKG2D receptor can sense DNA damage in host cells (100). Importantly, dysbiosis has been causally liked with DDR activation, *via* either the release of genotoxin or by promoting chronic inflammation (92). Several questions about the causal link of dysbiosis with NKG2D-Ls induction *via* DDR remain unanswered. For example, the precise mechanisms by which DDR induced by bacterial genotoxins or dysbiosis may result in malignant transformation or autoimmunity have not been fully elucidated. In addition, the specific role of dysbiosis in the upregulation of NKG2D-Ls in the intestinal epithelial cells of patients with IBD needs to be clarified. The polymorphism rs1049174 in the NKG2D gene (generating HNK and LNK genotypes) influences NKG2D receptor expression on immune cells and is implicated in individual susceptibility to certain cancers. The HNK genotype is associated with greater NK cells cytotoxic activity and lower prevalence of epithelial cells-derived malignancies, in comparison with the low cytotoxic genotype LNK (23, 50, 101, 102). It is currently unknown if HNK and LNK genotypes can affect NKG2D receptor-mediated immune responses in the context of dysbiosis and if they are implicated in the development of autoimmune disorders like IBD. Future studies on this regard are warranted as genetic variants of human genes involved in immunity and gut architecture are associated with an altered composition of the gut microbiome (103).

Compelling evidence indicate that dysbiosis is implicated in the pathogenesis of several human diseases, ranging from metabolic disorders, autoimmunity, and cancer (64, 104, 105) and various studies have shown that manipulating human microbiota, for example by using probiotics or fecal transplantation, has promising therapeutic potential (65, 106, 107). Further research is needed to uncover the specific microbes within a dysbiotic microbiota that are directly implicated in disease etiology. Designing optimal interventions aimed to remove pathogenic microorganisms or for replacing them with beneficial ones will have enormous therapeutic potential.

## Author Contributions

The authors contributed extensively to the work presented in this paper. JE: conceived and designed the study; created and drew figures and wrote the manuscript. MM: wrote the manuscript, searched and collected bibliography.

## Conflict of Interest Statement

The authors declare that the research was conducted in the absence of any commercial or financial relationships that could be construed as a potential conflict of interest.
